# Effect of Diet Supplementation on the Expression of Bovine Genes Associated with Fatty Acid Synthesis and Metabolism

**DOI:** 10.4137/bbi.s4168

**Published:** 2010-03-31

**Authors:** Sandeep J. Joseph, Kelly R. Robbins, Enrique Pavan, Scott L. Pratt, Susan K. Duckett, Romdhane Rekaya

**Affiliations:** 1 Rhodes Center for Animal and Dairy Science; 2 Department of Statistics,; 3 Institute of Bioinformatics, University of Georgia, Athens, Georgia, 30602, USA; 4 Department of Animal and Veterinary Sciences, Clemson University, Clemson, SC, 29634, USA; 5 Instituto Nacional de Tecnología Agropecuaria, Balcarce, Argentina.

**Keywords:** affymetrix bovine array, fatty acid metabolism, RT-qPCR, conjugated linoleic acid, stearoyl-CoA desaturase, fatty acid synthase, acetyl-coenzyme A carboxylase

## Abstract

Conjugated linoleic acids (CLA) are of important nutritional and health benefit to human. Food products of animal origin are their major dietary source and their concentration increases with high concentrate diets fed to animals. To examine the effects of diet supplementation on the expression of genes related to lipid metabolism, 28 Angus steers were fed either pasture only, pasture with soybean hulls and corn oil, pasture with corn grain, or high concentrate diet. At slaughter, samples of subcutaneous adipose tissue were collected, from which RNA was extracted. Relative abundance of gene expression was measured using Affymetrix GeneChip Bovine Genome array. An ANOVA model nested within gene was used to analyze the background adjusted, normalized average difference of probe-level intensities. To control experiment wise error, a false discovery rate of 0.01 was imposed on all contrasts. Expression of several genes involved in the synthesis of enzymes related to fatty acid metabolism and lipogenesis such as stearoyl-CoA desaturase (SCD), fatty acid synthetase (FASN), lipoprotein lipase (LPL), fatty-acyl elongase (LCE) along with several trancription factors and co-activators involved in lipogenesis were found to be differentially expressed. Confirmatory RT-qPCR was done to validate the microarray results, which showed satisfactory correspondence between the two platforms. Results show that changes in diet by increasing dietary energy intake by supplementing high concentrate diet have effects on the transcription of genes encoding enzymes involved in fat metabolism which in turn has effects on fatty acid content in the carcass tissue as well as carcass quality. Corn supplementation either as oil or grain appeared to significantly alter the expression of genes directly associated with fatty acid synthesis.

## Introduction

Food products of animal origin contribute significantly to the total nutrients in the human diet. Food products from ruminants are the major dietary source of conjugated linoleic acids (CLA) that are important for human health. The cis-9, trans-11 CLA isomer has been suggested to be an anticarcinogen, which reduced tumor proliferation in mice with experimentally induced epidermal carcinogenesis.^1^ Cis-9, trans-11 CLA isomer and trans-11 vaccenic acid (TVA) are produced in the rumen as intermediates in the biohydrogenation of dietary linoleic acid to stearic acid.^2^ Since only a small amount of the cis-9, trans-11 CLA is available for absorption in the small intestine, the major source of this CLA isomer in the adipose tissue of ruminants is from the conversion of trans-11 vaccenic acid to cis-9, trans-11 CLA in the adipose tissue by stearoyl-CoA desaturase (SCD).^3^ Noci et al^4^ observed a linear relationship between trans-vaccenic acid and cis-9, trans-11 CLA in the fat of beef cattle. Daniel et al^5^ concluded that high concentrate diets increase *SCD* gene expression in comparison to forage diets and suggested that the increase in cis-9, trans-11 CLA with forage diets would be due to an increase in the amount of TVA coming from the rumen. Thus, one way to increase cis-9, trans-11 CLA in beef products would be to increase production of TVA in the rumen by supplementing cattle with high levels of lipids rich in linoleic acids. However this type of supplementation would also increase the proportion of polyunsaturated fatty acids (PUFA) in beef fat^6^ and PUFA have been described to depress *SCD* gene expression. In addition, lipid supplementation could reduce lipogenesis by reducing enzyme activity, enzyme gene expression, or both, where as supplementation with glucogenic precursors (grains) would have the opposite effect.^7^ Recently, Pavan et al^8^ and Pavan and Duckett^9^ found that supplementation of corn oil to high concentrate diets or grazing cattle increases carcass fatness and alters fatty acid composition of adipose tissues including cis-9 trans-11 CLA.

According to Pegorier et al^10^ there is evidence suggesting that dietary constituents such as fatty acids and glucose regulate gene expression in humans. Such studies have also been done in ruminants, where Bonnet et al^11^ found that lipoprotein lipase activity and mRNA are up regulated in adipose tissues and cardiac muscle of sheep that were re-fed (190% of metabolizable energy requirement) after a period of under-feeding (7 d at 22% of metabolizable energy requirement). Loor et al^12^ studied the changes in hepatic gene expression using longitudinal transcript and metabolic profiling. Lehnert et al^13^ used cDNA microarrays for studying the effects of chronic and severe under-nutrition on the gene expression of bovine skeletal muscles. Microarray technology has been widely used for genome-wide expression profiling of thousands of genes that help bovine researchers to monitor genetic mechanisms regulating a variety of preferred traits involving disease resistance, nutrient partitioning, mammary development, muscle growth and stress tolerance. 14 Knowledge of the genes involved in digestion, absorption, and nutrient metabolism would allow researchers to develop optimal nutritional regimes for cattle of particular genetic backgrounds reared in a variety of environments. Such genomic information could be used to develop breeding strategies that promote animal health, well-being and food safety. The objective of this trial was to determine the effect of isoenergetic supplementation with different energy sources on the transcription of genes involved in fat metabolism at subcutaneous adipose tissue of grazing beef steers.

## Materials and Methods

### Feeding trials

Twenty-eight Angus steers (289 ± 3.8 kg) were randomly assigned to four dietary treatments. Dietary treatments included 2 isocaloric supplementation treatments to steers grazing endophyte-free tall fescue, corn grain (**PC**; 0.52% of body weight [BW]) or corn oil (**PO**; 0.10% of BW) plus soybean hulls (0.45% of BW soybean hulls) as a carrier, no supplementation to steers grazing endophyte-free tall fescue (**P**), or a high concentrate diet (**C**; 80% corn grain, 2.6% soybean meal, 2.4% minerals, and 15% Bermuda grass hay). Steers on PC, PO and P were managed together under a rotational grazing system for 197 d; where as, C steers were maintained on an adjacent tall fescue pasture for the initial 105 d and then fed a high concentrate diet for 92 d. Additional information on experimental design, diet composition, animal performance and carcass quality is provided in Pavan and Duckett.^9^

### RNA isolation

At slaughter, samples of s.c. adipose tissue (head tail) were obtained immediately after exsanguination, frozen in liquid nitrogen and then stored at −60 ºC. Several research studies have shown that nutritional effects on fatty acid composition are similar among intramuscular and subcutaneous adipose tissue.^9^,^15^,^16^ Both depots are actively being deposited during the finishing phase as evidenced by increases in external and marbling fat and the enzyme levels are similar between the two depots as well.^17^ Moreover RNA must be extracted from the tissue as soon as possible in order to have high quality RNA and s.c adipose tissue makes this task easier.

Total cellular RNA (tcRNA) was extracted from the s.c. adipose tissue (2 g) using TRIzol reagent (Gibco Invitrogen, Carlsbad, CA) according to manufacturer’s protocol. Following tcRNA extraction, Qiagen RNEasy MinElute Cleanup Kits were used for RNA cleanup, and a Quant-IT kit (Invitrogen, Carlsbad, CA) was used for RNA quantification. Purity and integrity of isolated RNA was confirmed by visualization of 18 S and 28 S ribosomal bands of individual samples subjected to denaturing slab gel electrophoresis in 1.2% ethidium bromide-stained agarose gel and 260/280 absorbance ratios. All tcRNA samples used in this experiment exhibited 260:280 ratios > 1.8.

### Microarray

For microarray analysis, tcRNA was pooled by treatment on an equal weight basis for all individual samples in a treatment group. The Affymetrix GeneChip Bovine Genome array was used for hybridization of the extracted mRNA’s according to the Affymetrix protocol at Medical College of Georgia, Genomics facility. The GeneChip Bovine Genome Array contained 24,027 probe sets, representing over 23,000 transcripts. The array was scanned and the gene expression data was generated using the Affymetrix software.

### Statistical analysis of microarray data

Statistical analysis was performed for each gene separately using the following simple linear model:

$$y_{ijk}=\mu_{i}+T_{ij}+e_{ijk}$$

where *y_ijk_* is the base-2 logarithm of background adjusted and normalized average difference of signal intensity for gene *i* in array *k*; *μ_i_* is the overall mean expression of gene *i*, *T_ij_* is dietary supplement *j* (*j* = 1,2,3,4) on gene *i*, and *e_ijk_* is the residual term.

F-ratios were computed for all possible contrasts between the treatment levels using the contrast statement in SAS. In order to control experiment wise error rates, a false discovery rate (FDR) criteria was employed. The Benjamini Hochberg method^18^ was used to identify genes differentially expressed at a given FDR level. The process is summarized in the following steps:

1. After selecting a significance level, α_e_, genes were sorted by *P*-values from the most to the least significant.
2. Threshold values were calculated for each test as:
  $$threshold_{i}=\frac{i*\alpha_{e}}{R}\text{for i }=1\;\text{to R}$$
  where R is the total number of tests (also the total number of genes) and i is the sorted position of the test.
3. Each *P*-value was then compared to its respective threshold, starting with the most significant *P*-value, until a *P*-value greater than the threshold value was encountered. All following contrast was then considered to be insignificant. For this study an FDR of 0.01 was used to identify potentially differentially expressed genes.

Least square estimates of both treatments for each single gene was calculated using the LSMEANS statement of SAS. The fold-change for each gene was calculated by taking the ratio of the least square estimates for all the four treatment comparisons to determine the magnitude of change in gene activity corresponding to each diet supplement.

### Relative quantification of lipogenic gene expression by Real-time PCR

PCR primers that span intron/exon junctions were designed using Primer3 software.^19^ Primer sequences for both end-point and real time quantitative PCR efficiencies are presented in Table 1. Primer sets were evaluated first by using end-point PCR. Pooled tcRNA (1 Âμg) was reverse-transcribed in a 20 Âμl reaction volume using oligo dT and SuperScript ™ III reverse transcriptase (Invitrogen) in a 2-step RT-qPCR reaction. PCR was conducted using GoTaq (Promega, Madison, WI) and 100 nM of each respective primer. Products were subjected to slab gel electrophoresis and visualized by EtBr staining and fluorescence. Further all products were purified and subjected to di-deoxy sequencing at the Clemson University Genomics Institute and database comparisons made to verify identity of PCR products. For real time quantitative PCR (qPCR), pooled tcRNA (1 Âμg) was reverse transcribed as stated above. A standard curve based on the original mass of tcRNA in the RT reaction was generated (50, 10, 2, 0.4, 0.08, and 0.016 ng per reaction) ran in triplicate, and subjected to qPCR by using the QuantiTect ^®^ SYBR^®^ Green PCR kit (Qiagen) on an Eppendorf^®^ Mastercycler ^®^ ep realplex (Eppendorf). Primer efficiency was calculated by regression analyses using the Eppendorf Mastercycler Software and all efficiencies were between 0.85–1.07. For all qPCR conducted the thermal cycling conditions included; DNA polymerase activation at 95 °C for 15 min., 40 PCR cycles for 15 s at 94 °C, 30 s at 60 °C, and 30 s at 72 °C in the presence of 100 nM of each primer combination.

### Procedure and statistical analysis of qRT-PCR

One Âμg of tcRNA for each individual animal was reverse transcribed and the quality of the RT reaction was evaluated by end-point PCR using the GAPDH primer set. Quantitative PCR was then conducted on 2 ng of the RT reaction with each primer set combination for all samples. The transcript levels for each gene were calculated at cycle threshold values (C_q_) at which each fluorescent signal was first detected above background. Two genes, GAPDH and β-actin, were evaluated as housekeeping genes for data normalization. To determine the appropriate housekeeping gene to be used to normalize the data, the C_q_ for GAPDH and β-actin, and all target genes per sample were entered into the BESTKEEPER program.^20^ This program determines the most stable housekeeping gene to be used for normalization by repeated pair-wise correlation analysis. Both GAPDH and β-actin exhibited a coefficient of correlation of 0.99 (*P* < 0.001) and were considered suitable for data normalization. For the analysis of relative gene expression, all C_q_ values for each sample/primer pair combination and the respective primer efficiency were analyzed using the REST-2008 V2.0.7 program (http://www.gene-quantification.de/rest-2008.html)^21^ and the data was normalized using GAPDH. This software calculates relative gene expression using Pair-wise Fixed Reallocation Randomization Test and relative expression determined at the 95% confidence interval.

## Results and Discussion

Several genes relevant to lipid metabolism were found to be differently expressed in all four-treatment comparisons. The number of genes found to be differentially expressed when using an FDR of 0.01 can be found in Table 2. In this study, the largest number of differentially expressed genes was observed for the contrast between P and C (1113 genes), which shows potential evidence of altered gene expression on animal with diet supplementation. Andrae et al^22^ reported that high oil intake increases the energy density of the diet and this will alter the pattern of lipid deposition in steers fed with finishing diet. This change in lipid deposition could be due to changes in gene expression and this process could be inferred from the comparison between PO and C fed animals as well as between PO and P. In fact, 757 and 89 genes were differentially expressed between PO and C and PO and P, respectively.

**Figure 1:**
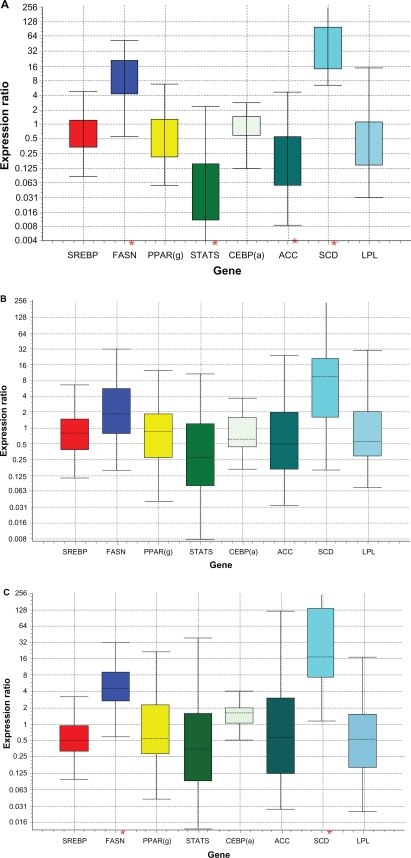
Relative expression from qRT-PCR analysis. **A**) Indicates the relative expression of C (Concentrate, corn grain) with respect to P (Pasture). **B**) Indicates the relative expression of PO (Pasture and Corn oil) with respect to P (Pasture). **C**) Indicates the relative expression of PC (pasture and corn grain) with respect to P (Pasture). Boxes represents the interquartile range, or the middle 50% of observations. The dotted line represents the median gene expression. Whiskers represent the minimum and maximum observations. The asterisk (*) represents genes that are significantly differentially expressed.

The *P*-value of genes related to lipogenesis and gluconeogenesis can be found in Tables 3 and 4, respectively. Stearoyl-CoA desaturase (SCD) was viewed as a key lipogenic enzyme not only for its role in the conversion of saturated fatty acid (SFA) to monounsaturated fatty acids (MUFA) by inserting a double bond in Δ^9^ position but also for its pattern of regulation by diet supplementation. In our microarray gene expression analysis, SCD was found to be differentially expressed, between P and PC (*P* < 0.01), PO and PC (*P* < 0.01), PC and C (*P* < 0.01) and P and C (*P* < 0.01). Furthermore, expression of SCD was up-regulated in animals supplemented with corn grain (C) compared to those which were on grazing pasture (P) or pasture and corn grain (PC). However, the same gene was not significantly differentially expressed in comparisons between P and PO or C and PO (Table 3). The confirmatory gene expression analysis using qRT-PCR showed that SCD mRNA expression was up-regulated by 47- (*P* < 0.001), 30- (*P* = 0.002) fold, respectively for C and PC compared to P, while PO was only up-regulated by 7- fold (*P* = 0.068) compared to P (Tables 6, 7 and 8; Fig. 1(A), 1(B) and 1(C)). The most important result to be noticed from qRT-PCR analysis for SCD gene when compared between P and PO (Table 9) was that there were no differences in the relative abundance of SCD mRNA between the two treatments, which were similar with microarray results. Chung et al^23^ reported increases in SCD activity of s.c adipose tissue of Angus and Wagyu steers fed with grain compared to hay; however changes in SCD mRNA were dependent upon breed type, with increased mRNA level in Wagyu. Daniel et al^5^ also reported higher SCD expression and increased *MUFA* in ovine adipose tissue explants exposed to insulin in vitro. Our data suggest that corn grain significantly increased the transcription of the SCD gene while corn oil supplementation resulted in a lower increase in MUFA and SCD expression, which was not significantly different, compared to P for both microarray and qRT-PCR analysis. Further studies^24^ regarding the effects of these supplementary diets on the subcutaneous fatty acid content using the same experimental animals showed that corn oil (PO) supplementation increased TVA and cis-9, trans-11 CLA isomer content in subcutaneous tissue relative to P, PC and C. This suggests that supplementation of corn oil increased PUFA that affects the expression of SCD. As a result, it can be inferred that the presence of corn oil or grain in the diet may alter the expression of genes important to stearoyl-coenzyme A desaturase; which in turn, may influence fatty acid synthesis.

Fatty acid synthase (FASN), which is thought to be the rate-limiting step in de novo fatty acid synthesis in ruminants was differentially expressed between PO and C (*P* < 0.01), PC and C (*P* < 0.01), PO and P (*P* < 0.01) and P and C (*P* < 0.01). Table 3 shows that there is an increased gene expression of FASN in animals fed with corn grain (C) compared to those fed with corn oil and pasture. This suggests that concentrate in the diet may increase fatty acid biosynthesis. Real time PCR confirmed this result (Table 6; Figure 1(a)) where the relative expression of FASN trends to be higher in animals fed with concentrate (C) diets when compared with those fed with pasture (P) only. Acetyl-coenzyme A carboxylase (*ACC)* mRNA expression did not differ among majority of the treatments in both gene expression analysis methods (Table 3). Both ACC and FASN are key enzymes regulating *de novo* fatty acid synthesis. Grain feeding, either on a high-concentrate diet or supplemented on pasture, up-regulated FASN mRNA compared to P. Up-regulation of FASN with grain feeding would suggest greater *de novo* lipogenesis. The lack of significant differential expression in FASN mRNA with oil supplementation on pasture (PO) compared to PC (Table 3) indicates that corn supplementation and not an increased energy supplementation is responsible for the change in gene expression of FASN. Research in humans has shown that increased expression of FASN in adipose tissue is linked to excess fat accumulation and impaired insulin sensitivity.^25^ Geetha et al^26^ investigated the effects of exogenous fatty acid treatment on the function of *ACC* and *FASN* in mammary cells and found that, increasing concentrations of exogenous oleic acid and TVA caused inhibition of ACC and FASN activities. Among the two monounsaturated fatty acids, TVA was much more powerful inhibitor of ACC and FASN, when compared with oleic acid. Similar effects are also observed in this study, as the gene expression of FASN (Table 3) in adipose tissue has been found to be lower in corn oil fed animals (C and PO) because corn oil does contain oleic acid (28.5% of total fatty acids in corn oil).

Lipoprotein lipase (LPL), a lipolytic enzyme involved in the metabolism of triacylglycerol-rich lipoprotein particles, which generate free fatty acids, was found to be differentially expressed between P and PC (*P* < 0.01), C and PO (*P* < 0.01) and C and PC (*P* < 0.01). While LPL was not found to be differentially expressed between P and C as well as P and PO, which shows correspondence with the results obtained from qRT-PCR (Tables 3 and 9). This enzyme has the direct opposite function of FASN, which mediates the synthesis of fatty acids.

The gene that codes for long-chain fatty-acyl elongase (LCE) enzyme was also differentially expressed between C and PO (*P* < 0.01) and C and P (*P* < 0.01) (Table 3). Moon et al^27^ suggested that mouse LCE expression is increased by sterol regulatory element-binding proteins (SREBPs) and that the enzyme is a component of the mammalian elongation system that converts palmitic to stearic acid by enhancing the addition of 2-carbon units to palmitic acid. LCE promotes increased production of stearic acid which is a saturated fatty acid. Our results indicate that there was an increased relative expression of LCE in animals fed with corn grain than with pasture or corn oil, suggesting that corn grain increases the production of saturated fatty acids in ruminants.

Furthermore, the glycerol phosphate acyltranferase (GPAT) gene which is involved in synthesis of triglycerides was found to be differentially expressed in almost all the dietary treatment comparisons. Several other genes responsible for the production of fatty acid-binding proteins were identified as differentially expressed in P vs. PC and C vs. PC comparisons. Studies by Peterson et al^28^ found a coordinated change in the mRNA abundance for genes that code for ACC, FASN and GPAT when they induced milk fat depression (MFD) with a high concentrate/low forage diet and examined the milk composition.

Genes responsible for the production of enzymes involved in the conversion of substrates to citric acid cycle intermediates (gluconeogenesis) are shown in Table 4. Potential genes involved in this process like propionyl—CoA carboxylase alpha (PCCA), methylmalonyl—CoA mutase (MUT), methylmalonyl—CoA epimerase (MCE), succinate dehydrogenase complex (SDH), phosphoenolpyruvate carboxylase 1 (PCK1), phosphoenolpyruvate carboxylase 2 (PCK2) and fructose—1,6-bisphosphate (FBP2) were found to be differentially expressed among the various treatment comparisons. PCCA, MCM, MCE and FBP2 genes were found to be down regulated in animals fed with corn oil compared to pasture fed animals. Propionyl—CoA carboxylase alpha enzyme plays a vital role in propionate metabolism, where it catalyses the intermediate step between propionyl-CoA and methylmalonyl CoA. Methylmalonyl—CoA mutase functions in propanoate metabolism where it activates the intermediate step between methylmalonyl—CoA and succinyl—CoA, where as succinate dehydrogenase complex catalyses the conversion of succinyl—CoA to succinate. The enzymes, PCK1 and PCK2 that plays an important role in pyruvate metabolism by catalyzing the conversion of phosphoenol pyruvate to oxaloacetate, was also expressed higher in animals fed with corn grain. It appears that corn oil may decrease expression of some gluconeogenic enzymes compared to those steers that were fed with non-supplemented feed, where as those steers fed with corn grain would have a higher expression of gluconeogenic enzymes with respect to the grazing treatments.

Regulation of fatty acid metabolism is done through changes in transcription, mRNA processing or activity of several transcription factors. *P*-value and fold change of the expression of genes for transcription factors or coactivators involved in lipogenesis from the microarray analysis are shown in Table 5. Peroxisome proliferator-activated receptors gamma (PPARγ) is a nuclear hormone receptor whose transcriptional activities are stimulated by ligands and plays a vital role in the induction of genes mediating fatty acid uptake, metabolism (involved in fatty acid oxidation) and storage.^29^ Another transcription factor, sterol regulatory element-binding protein-1 (SREBP-1) was found to be involved in fatty acid synthesis.^30^ CCCAAT/enhancer-binding protein alpha (CEBPα) is another transcription factor involved in regulating adipogenesis. Clarke et al^31^ found that CEBPα mediates the expression of PPARγ and thus both play a role in regulating the expression of other proteins necessary for the development of mature adipocyte. STAT5’s (signal transducer and activator of transcription), has been reported to function in fat-cell development, adipocyte differentiation and lipid accumulation by regulating PPARγ and CEBPα binding signals.^32^ Growth hormones and prolactin are activators of STAT5 and induce lipolysis.^33^ Hogan and Stephens^34^ have shown that STAT5 directly represses the expression of FASN in adipocytes. Havatine and Bauman^35^ reported reduced expression of FASN, LPL and SREBP-1 in mammary tissue of cows fed with trans-10 cis-12 CLA isomer or low forage/high oil diet to induce milk-fat depression. In the current study, LPL and SREBP mRNA levels were unchanged with corn oil dietary supplementation. This is consistent with the findings by Duran-Montge et al^36^ where they found that the mRNA expression of SREBP in adipose tissue in pigs showed no differences between various lipid dietary treatments, while they observed increase in the expression of liver SREBP mRNA. This suggests that fat synthesis in adipose tissue could be regulated in a different manner than in liver. Additional research has shown that mRNA in adipose tissue of FASN, LPL and SREBP are not down regulated until dietary corn oil supplementation levels reach 0.15% of BW, levels higher than that were fed in this study (Duckett and Pratt, unpublished data). PPAR(γ) and CEBP(α) in microarray analysis were found to be differentially expressed in the comparison between P and PC with a reduced gene activity in PC, where as RT-PCR results (Fig. 1c) of the same comparison confirmed the microarray detection of PPARγ, but the gene expression for both were found to be unchanged. PPAR(γ) and CEBP(α) were found to be differentially expressed in microarray analysis between PO and PC with increased gene activity in PO and in all other comparisons (P and PO, C and PO, C and PC and C and P) they were not found differentially expressed (Table 5) as well in the confirmatory qRT-PCR analysis (Tables 6,7 and 8).

Validation of high throughput methods like microarray using qRT-PCR has become a routine procedure and it is supposed to be the strongest confirmatory detection method done independently to quantify gene expression. Although Affymetrix arrays show high precision and repeatability, array results can be influenced by the variation in manufacturing process, sample preparation and data analysis. In the current study, eight genes (that have an influence in lipid metabolism based on previous studies) were selected to conduct qRT-PCR analysis to confirm our results obtained from microarray. The highest correspondence between microarray and qRT-PCR results were obtained between the comparison of P versus PO (7 out of 8) and P versus C (6 out of 8 genes) validated using qRT-PCR. While for P versus PC the microarray results of 4 genes showed correspondence with the confirmatory qRT-PCR results (Table 9). This slight difference in the correspondence across the two platforms might be accounted due to the pooling of RNA for microarray analysis where as individual RNA samples were used for qRT-PCR. Also, the qRT-PCR primer is designed based on the sequence database, which is derived from multiple sequence alignment of ESTs. As a result there is a high chance that affymetrix may target one gene, while the sequence database may infact represent an entire gene family with less specificity and therefore will interrogate neither the Affymetrix gene nor the database gene and thus give results that differ from both platforms.^37^

Despite the changes in gene expression, Pavan et al^38^ found that there were significant effects on the carcass qualities with these dietary supplements. Carcass traits did not differ between PC and PO while the dressing percentage and Hot Carcass Weight (HCW) where greater in PC, PO and C compared to P. Marbling score, Longissimus Muscle (LM) area and quality grade did not differ between grazing treatment. Pavan et al^38^ observed that the energy supplementation (PC and PO) increased the dressing percentage and carcass weight compared to P however these steers has less dressing percentage and carcass weight compared with steers supplemented with C. All these suggest that the effects on the expression of genes in fatty acid metabolism by high concentrate diet increases the fatty acid metabolism, which in turn is also reflected in carcass quality.

## Conclusions

Maintenance of energy homeostasis occurs through the induction of genes coding for enzymes that regulate specific rate limiting steps in lipid and energy metabolism. Consequently, the metabolic effects of CLA are presumed to involve changes in gene expression. Control of lipid homeostasis in response to the body’s energy requirements is primarily exerted through transcription factors of the nuclear hormone receptor family. These receptors bind small, lipophilic molecules that modulate receptor activation state. The results of this study showed that diet composition could have a significant impact on the expression of several genes crucially important to fatty acid and lipid metabolism. Corn supplementation either as oil or grain appeared to significantly alter the expression of genes directly associated with fatty acid synthesis. Based on previous studies, our results suggest that the effects of gene expression on different concentrate dietary supplements on adipose tissue are different from those found in other tissues. These results were confirmed with qRT-PCR that showed satisfactory correspondence across the two platforms.

## Figures and Tables

**Table 1:** Primer sequences (5′ to 3′) for quantitative real-time PCR.

**Gene**	**Forward**	**Reverse**	**Efficiency**
β-Actin	CTCTTCCAGCCTTCCTTCCT	GGGCAGTGATCTCTTTCTGC	0.85
ACC	AGCTGAATTTTCGCAGCAAT	GGTTTTCTCCCCAGGAAAAG	1.07
C/EBPα	TGGACAAGAACAGCAACGAG	GGTCATTGTCACTGGTCAGC	0.95
FASN	GCATCGCTGGCTACTCCTAC	GTGTAGGCCATCACGAAGGT	0.93
GAPDH	GGGTCATCATCTCTGCACCT	GGTCATAAGTCCCTCCACGA	0.93
LPL	GGGTTTTGAGCAAGGGTACA	GCCACAATGACCTTTCCAGT	0.97
PPARγ	AGGATGGGGTCCTCATATCC	GCGTTGAACTTCACAGCAAA	0.86
SCD	TTATTCCGTTATGCCCTTGG	GGTAGTTGTGGAAGCCCTCA	0.95
Spot14	CCTCACCCATCTTACCCTGA	CAAGCTAGCAAACTGCACCA	1.05
SREBP1	CTGGAGAAGCTGGACTGAGG	GCTTTCCCAAGACTCAGCAC	0.86
STAT5	TGGGAAAGATGGGAACTGAG	ACCAACAAGTCTGGGTCAGG	1.00

**Table 2:** Number of genes differentially expressed between the four treatments.^A^

**Treatments**	**P**	**C**	**PC**	**PO**
P	0	1113	39	89
C	1113	0	113	757
PC	39	113	0	183
PO	89	757	183	0

**Abbrivations:**

A P, pasture only; PO, pasture and corn oil; C, concentrate; PC, pasture and corn grain.

**Table 3:** Enzymes involved in lipogenesis differentially expressed among the dietary four treatments in microarray analysis and their *P*-value.^B^

**Gene**	**Gene product**	**P vs. PO**	**P vs. PC**	**PO vs. PC**	**C vs. PO**	**C vs. PC**	**C vs. P**
ACC	Acetyl-coA carboxylase	0.11	0.09	0.02	0.16	0.11	<0.01* (4.62)↑
FASN	Fatty acid synthase	<0.01* (−0.70)↓	0.01	0.01	<0.01* (0.71)↑	<.01* (1.67)↑	<0.01* (0.52)↑
SCD	Stearoyl-coA desaturase	0.51	<0.01* (4.44)↓	<0.01* (4.256)↑	0.09	<0.01* (3.96)↑	<0.01* (6.48)↑
LCE	Long-chain fatty-acyl elongase	0.43	0.13	0.39	<0.01* (0.64)↑	0.05	<0.01* (4.067)↑
IDH1	Isocitrate dehydrogenase 1 (NADP+), soluble	0.14	<0.01* (4.12)	<0.01* (3.72)↑	<0.01* (0.89)↑	<0.01* (2.91)↑	<0.01* (2.00)↑
6-PGD	6-phosphogluconate dehydrogenase	0.08	0.03	0.01	<0.01* (0.522)↑	<0.01* (2.36)↑	<0.01* (1.50)↑
LPL	Lipoprotein lipase	0.75	<0.01* (1.65)↑	0.02	<0.01* (0.183)↑	<0.01* (2.39)↑	0.10
GPAT	Glycerol-3-P acyltransferase, mitochondrial	<0.01* (−1.18)↓	<0.01* (−7.35)↓	<0.01* (−6.170)↓	<0.01* (0.616)↑	<0.01* (5.54)↑	<0.01* (1.80)↑
DGAT2	Putative Diacylglycerol O-acyltransferase	<0.01* (−0.524)↓	0.03	<0.01* (3.295)↑	<0.01* (1.37)↑	<0.01* (1.978)↑	<0.01* (1.84)↑
SLC25 A	Solute carrier family 25 (citrate transporter)	<0.01* (−2.453)↓	0.89	0.08	<0.01* (1.72)↑	<0.01* (0.120)↑	<0.01* (4.181)↑

B,* indicates the *P*-values of significantly differentially expressed genes using a false discovery rate of 0.01.

^↓^indicates lower expression in A than in B (A vs. B); ^↑^indicates higher expression in A than in B (A vs. B).

Values inside the parenthesis are the fold change.

**Abbreviations:** P, pasture only; PO, pasture and corn oil; C, concentrate; PC, pasture and corn grain.

**Table 4:** Enzymes involved in the conversion of substrates to citric acid cycle intermediates (gluconeogenesis) that were differentially expressed among the four dietary treatments in microarray analysis and their *P*-value.^C^

**Gene**	**Gene product**	**P vs. PO**	**P vs. PC**	**PO vs. PC**	**C vs. PO**	**C vs. PC**	**C vs. P**
PCCA	Propionyl-coA carboxylase alpha	<0.01* (−0.32)↓	0.67	0.26	<0.01* (0.5737)↑	0.14	<0.01* (0.897)↑
MCM	Methylmalonyl-coA mutase	<0.01* (−1.33)↓	0.02	0.08	<0.01* (0.589)↑	0.01	<0.01* (1.982)↑
MCE	Methylmalony-coA epimerase	<0.01* (−0.82)↓	0.02	0.01	<0.01* (−0.223)↑	<0.01* (2.14)↑	<0.01* (1.56)↑
SDHc	Succinate dehydrogenase complex (C)	0.07	0.11	0.05	<0.01* (0.387)↑	0.01	<0.01* (1.09)↑
SDHd	Succinate dehydrogenase complex (D)	0.32	<0.01* (5.927)↑	<0.01* (5.10)↑	<0.01* (0.56)↑	<0.01* (5.035)↑	<0.01* (0.891)↑
PCK1	Phosphoenolpyruvate carboxylase 1	0.17	0.18	0.07	0.48	<0.01* (0.807)↑	<0.01* (0.884)↑
PCK2	Phosphoenolpyruvate carboxylase 2	0.05	0.10	0.01	0.02	0.02	<0.01* (2.437)↑
FBP2	Fructose-1, 6-bisphosphate	<0.01* (−2.40)↓	0.12	0.03	<0.01* (−0.52)↓	0.31	<0.01* (−1.87)↓

C,* indicates the *P*-values of significantly differentially expressed genes using a false discovery rate of 0.01;

^↓^indicates lower expression in A than in B (A vs. B); ^↑^indicates higher expression in A than in B (A vs. B).

Values inside the parenthesis are the fold change.

**Abbreviations:** P, pasture only; PO, pasture and corn oil; C, concentrate; PC, pasture and corn grain.

**Table 5:** Genes for transcription factors or coactivators involved in lipogensis differentially expressed among the four dietary treatments in microarray analysis and their *P*-value.^D^

**Gene**	**Transcription factor**	**P vs. PO**	**P vs. PC**	**PO vs. PC**	**C vs. PO**	**C vs. PC**	**C vs. P**
PPAR(γ)	Perxoisome proliferators activated Receptor (gamma)	0.1129	<0.01* (3.9110)↑	<0.01* (5.11)↑	0.8011	0.032	0.175
STAT5B	Signal transducer and activator of transcription 5B	0.9877	0.2260	0.375	0.090	0.2430	0.1032
SREBP	Sterol regulatory element binding protein	0.8901	0.150	0.2675	0.9644	0.2554	0.831
CEBP(α)	CCAAT/enhancer—binding protein (alpha)	0.2739	<0.01* (4.647)↑	0.011	0.152	<0.01* (3.37)↑	0.931

D,* indicates the *P*-values of significantly differentially expressed genes using a false discovery rate of 0.01;

↓ indicates lower expression in A than in B (A vs. B); ^↑^indicates higher expression in A than in B (A vs. B).

Values inside the parenthesis are the fold change.

**Abbreviations:** P, pasture only; PO, pasture and corn oil; C, concentrate; PC, pasture and corn grain.

**Table 6:** Relative gene expression obtained for C (concentrate, treatment) with respect to P (pasture, control) using RT-PCR.^E^

**Gene**	**Type**	**Expression**	**Std. error**	**95% C.I.**	**P(H1)**	**Result**
GAPDH	REF	1.000				
SREBP	TRG	0.593	0.208–1.684	0.110–3.203	0.239	NR
FASN	TRG	8.849	2.805–30.023	0.931–49.909	0.006	UP
PPARγ	TRG	0.540	0.121–2.243	0.059–5.170	0.266	NR
STAT5	TRG	0.051	0.007–0.408	0.002–1.642	0.007	DOWN
CEBPα	TRG	0.862	0.372–1.753	0.165–2.752	0.687	NR
ACC	TRG	0.181	0.039–1.415	0.012–3.720	0.042	DOWN
SCD	TRG	47.140	11.700–447.69	6.843–2,194.549	<0.0001	UP
LPL	TRG	0.485	0.087–2.153	0.046–9.564	0.283	NR

E P(H1)—Probability of alternate hypothesis that difference between sample (C) and control groups (P) is due only to chance.

**Abbreviations:** TRG, Target; REF, Reference; NR, not significantly regulated.

**Table 7:** Relative gene expression obtained for PO (corn oil and pasture, treatment) with respect to P (pasture, control) using RT-PCR.^F^

**Gene**	**Type**	**Expression**	**Std. error**	**95% C.I.**	**P(H1)**	**Result**
GAPDH	REF	1.000				
SREBP	TRG	0.809	0.321–1.933	0.147–4.362	0.602	NR
FASN	TRG	2.218	0.572–18.831	0.219–30.941	0.283	NR
PPARγ	TRG	0.820	0.182–4.452	0.063–9.497	0.748	NR
STAT5	TRG	0.333	0.051–2.477	0.012–7.607	0.181	NR
CEBPα	TRG	0.815	0.310–1.998	0.186–3.713	0.590	NR
ACC	TRG	0.608	0.093–4.004	0.047–15.540	0.489	NR
SCD	TRG	7.187	0.484–68.919	0.213–1,036.416	0.068	NR
LPL	TRG	0.892	0.188–3.174	0.113–20.060	0.847	NR

F P(H1)—Probability of alternate hypothesis that difference between sample (C) and control groups (P) is due only to chance.

**Abbreviations:** TRG, Target; REF, Reference; NR, not significantly regulated.

**Table 8:** Relative gene expression obtained for PC (corn grain and pasture, treatment) with respect to P (pasture, control) using RT-PCR.^G^

**Gene**	**Type**	**Expression**	**Std. error**	**95% C.I.**	**P(H1)**	**Result**
GAPDH	REF	1.000				
SREBP	TRG	0.533	0.177–1.596	0.111–2.865	0.139	NR
FASN	TRG	4.575	1.721–11.813	0.843–30.280	0.006	UP
PPARγ	TRG	0.643	0.099–3.015	0.046–15.982	0.505	NR
STAT5	TRG	0.387	0.051–2.017	0.020–25.991	0.268	NR
CEBPα	TRG	1.558	0.841–3.126	0.618–3.970	0.089	NR
ACC	TRG	0.640	0.070–5.163	0.036–73.163	0.614	NR
SCD	TRG	30.314	2.661–245.852	1.407–1,527.9	0.002	UP
LPL	TRG	0.566	0.094–3.073	0.039–11.392	0.418	NR

G P(H1)—Probability of alternate hypothesis that difference between sample (C) and control groups (PC) is due only to chance.

**Abbreviations:** TRG, Target; REF, Reference; NR, not significantly regulated.

**Table 9:** Comparison of Microarray and qRT-PCR results.^G^

**Transcript**	**Microarray**	**RT-PCR**	**Acceptable^**	**Correspondance**
	***P*-value$**	***P*-value#**		
**P vs. C**				
SREBP	0.831	0.239	Yes	6/8
PPARγ	0.175	0.266	Yes	
STAT5	0.1032	<0.01^*^(0.051)**↑**	No	
CEBPα	0.931	0.687	Yes	
ACC	<0.01^*^(4.62)**↓**	0.042^*^(0.181)**↑**	No	
SCD	<0.01^*^(6.48)**↓**	<0.01^*^(47.14)**↓**	Yes	
LPL	0.1	0.283	Yes	
FASN	<0.01^*^(0.52)**↓**	<0.01^*^(8.849)**↓**	Yes	
**P vs. PC**				
SREBP	0.15	0.139	Yes	4/8
PPARγ	<0.01^*^↑	0.505	No	
STAT5	0.226	0.268	Yes	
CEBPα	<0.01^*^(4.64)**↑**	0.089	No	
ACC	0.09	0.614	Yes	
SCD	<0.01^*^(3.96)**↓**	0.002^*^(30.31)**↓**	Yes	
LPL	<0.01^*^(1.65)**↓**	0.418	No	
FASN	<0.01	0.006^*^(4.57)**↑**	No	
**P vs. PO**				
SREBP	0.8901	0.6902	Yes	7/8
PPARγ	0.1129	0.748	Yes	
STAT5	0.9877	0.181	Yes	
CEBPα	0.2739	0.590	Yes	
ACC	0.11	0.489	Yes	
SCD	0.51	0.068	Yes	
LPL	0.75	0.847	Yes	
FASN	<0.01^*^(0.70)**↓**	0.283	No	

G, ↓indicates lower expression in A than in B (A vs. B); ^↑^indicates higher expression in A than in B (A vs. B).

* Indicates statistical significance;

$ FDR corrected *P*-values from the F-test done in microarray analysis.

# Probability (*P*-value) of alternate hypothesis that difference between samples (C, PC and PO) and control (P) groups are due only to chance.

^ indicates resemblance between microarray and qRT-PCR if Yes otherwise No.

**Abbreviations:** P, pasture only; PO, pasture and corn oil; C, concentrate; PC, pasture and corn grain.
